# Differences in residents’ self-reported confidence and case experience between two post-graduate rotation curricula: results of a nationwide survey in Japan

**DOI:** 10.1186/1472-6920-14-141

**Published:** 2014-07-12

**Authors:** Sachiko Ohde, Gautam A Deshpande, Osamu Takahashi, Tsuguya Fukui

**Affiliations:** 1 Center for Clinical Epidemiology, St Luke’s Life Science Institute, 10-1 Akashi-cho, 104-0044 Chuo-city, Tokyo, Japan; 2 Department of General Internal Medicine, St Luke’s International Hospital, 9-1 Akashi-cho, 104-8560 Chuo-city, Tokyo, Japan; 3 Department of Internal Medicine, University of Hawaii, Hawaii, USA

**Keywords:** Japanese junior residency education, Clinical competency

## Abstract

**Background:**

In Japan, all trainee physicians must begin clinical practice in a standardized, mandatory junior residency program, which encompasses the first two years of post-graduate medical training (PGY1 – PGY2). Implemented in 2004 to foster primary care skills, the comprehensive rotation program (CRP) requires junior residents to spend 14 months rotating through a comprehensive array of clinical departments including internal medicine, surgery, anesthesiology, obstetrics-gynecology (OBGYN), pediatrics, psychiatry, and rural medicine. In 2010, Japan’s health ministry relaxed this curricular requirement, allowing training programs to offer a limited rotation program (LRP), in which core departments constitute 10 months of training, with electives geared towards residents’ choice of career specialty comprising the remaining 14 months. The effectiveness of primary care skill acquisition during early training warrants evaluation. This study assesses self-reported confidence with clinical competencies, as well as case experience, between residents in CRP versus LRP curricula.

**Methods:**

A nation-wide cross-sectional study of all PGY2 physicians in Japan was conducted in March 2011. Primary outcomes were self-report confidence for 98 clinical competency items, and number of cases experienced for 85 common diseases. We compared confidence scores and case experience between residents in CRP and LRP programs, adjusting for parameters relevant to training.

**Results:**

Among 7506 PGY2 residents, 5052 replied to the survey (67.3%). Of 98 clinical competency items, CRP residents reported higher confidence in 12 items compared to those in an LRP curriculum, 10 of which remained significantly higher after adjustment. CRP trainees reported lower confidence scores in none of the items. Out of 85 diseases, LRP residents reported less experience with 11 diseases. CRP trainees reported lower case experience with one disease, though this did not remain significant on adjusted analysis. Confidence and case experience with OBGYN- and pediatrics-related items were particularly low among LRP trainees.

**Conclusions:**

Residents in the specialty-oriented LRP curriculum showed less confidence and less case experience compared to peers training in the broader CRP residency curriculum. In order to foster competence in independent primary care practice, junior residency programs requiring experience in a breadth of core departments should continue to be mandated to ensure adequate primary care skills.

## Background

In the rapidly specializing health care arena, the training of competent generalists remains a cornerstone of optimal population-level health as measured by a variety of outcomes in many countries [1]. Despite this understanding, implementation and prioritization of generalist education programs remains challenging [2]. Highlighting the gravity of these challenges, West et al. showed that, even amongst residents in a formal primary care residency training programs, more than half planned to pursue a subspecialty area of practice [3].

To adequately meet demand for generalist practitioners, it is crucial that young physicians receive adequate exposure to the full breadth of general practice. However, curricular structures that best meet this need, balancing core generalist competencies with specialized knowledge and skills, remain the subject of ongoing discussions amongst which curricular redesigns are not uncommon. To this end, in 2007, the Alliance for Academic Internal Medicine Education Redesign Task Force, sought to formalize a core set of knowledge, skills, and attitudes, proficiency in which should be maintained throughout a career, regardless of practice in a specialty or general medicine [4].

Formal residency training for recent medical school graduates was established more than 60 years ago in Japan. However, rules governing post-graduate training previously allowed the vast majority of young doctors to immediately begin training in their specialty department of choice, without mandating any period of time in either a general medicine or surgical curriculum. Nonetheless, due to the historical absence of formally trained primary care physicians in Japan, specialist trainees are expected to acquire skills needed to treat a wide variety of diseases common to primary care [5]. To better meet this need, the Japanese Ministry of Health established a 24-month, standardized comprehensive foundational curriculum (comprehensive residency program, CRP) that would serve to expose all young trainees to a wide variety of specialties, diseases, and skills. Exposure to the entire breadth of medical practice, even those areas not directly related to primary care, it was argued, would facilitate the development of well-rounded and comprehensively educated generalist physicians ready to tackle the growing primary care role as resource manager for both primary and secondary care [6] As political and professional controversy ensued, the CRP curriculum was relaxed and, in 2010, programs were allowed to implement a less comprehensive, specialty-oriented curriculum (limited residency program, LRP).

As of 2012, two different types of rotation programs exist in Japan, giving us a unique opportunity, one not found in other countries, to directly assess the efficacy of generalist training using two competing curricula offering a difference in breadth of training. Differences in training effectiveness of these two systems have not yet been assessed. The purpose of this study is to investigate PGY2 physicians’ self-reported confidence with knowledge, skills, and experience of diseases useful to primary care, and to compare these between residents trained in two different rotation curricula. We hypothesize that residents training in a more comprehensive program will be more confident and more experienced in regards to a variety of conditions seen in general practice compared to those residents’ opting for more limited curricula.

## Methods

### Context of the study

In Japan, specialty training is most often undertaken at university hospitals; 70% of residents in 2001 trained in a university hospital program, with only 30% belonging to an unaffiliated community hospital [7]. Of residents in university programs, 40% trained exclusively in one department, presumably the specialty of their future career. This training paradigm suggests that few residents were previously able to gain experience in the large variety of cases and skills required for the independent practice of primary care. Nonetheless, an estimated 33% of mid-career physicians in Japan eventually leave their hospital-based specialties to practice independent, primary care-oriented, community medicine [8].

Adult primary care residency curricula (family practice, general internal medicine) are relatively recent phenomena in Japan and are not widely offered as formal training tracks. Recognizing the need for adequate primary care training, the Ministry of Labor, Health, and Welfare (MLHW) of Japan implemented a mandatory junior residency program in 2004 with the purpose of fostering residents’ experience and skill in primary care, as well as enhancing professionalism and patient-centered practice. The CRP curriculum requires all 1^st^ - and 2^nd^-year (“junior”) residents to rotate through a standardized set of core departments comprised as follows: internal medicine subspecialties (6 months), surgery (3 months), anesthesiology (1 month), obstetrics-gynecology (OBGYN; 1 month), pediatrics (1 month), psychiatry (1 month), and rural/community medicine (1 month); the additional 10 months are generally spent in a variety of hospital-specific required rotations or career-oriented electives. As opposed to medical school clerkships in which Japanese students have limited patient contact [5,9], the CRP offers a hands-on experience similar to Transitional Residency in the US and Foundation Years in the UK education systems. One year after CRP implementation, national trends reflected that a majority of residents were choosing to do their junior residencies in general hospitals rather than university systems (50.8% versus 49.2%) [10]; four years later, self-reported confidence with a variety of diseases and practice competencies was also shown to have risen [11]. Historically, however, university hospitals have played a key role in physician allocation to rural areas; decreasing numbers of university trainees was perceived as exacerbating overall physician shortages to these underserved areas. In 2010, to accelerate the number of residents returning to university-based programs, Japan’s Ministry of Health allowed training programs to offer a limited rotation program (LRP), limiting the number of required core departments to less than one year, with remaining time spent in specialty-oriented selectives; junior LRP residents are required to rotate through internal medicine subspecialties (6 months), emergency medicine (3 months), and rural medicine (1 month). In addition, trainees are required to choose two selectives from surgery, OBGYN, pediatrics, psychiatry, or anesthesiology, though the period of time in selectives is neither specified nor regulated. The remainder of their two-year junior residency experience may be spent training in their specialty department of choice.

### Design, setting and sample

A nation-wide cross-sectional study of graduating PGY2 residents was conducted in Japan in March 2011, the end of the Japanese academic year. Participants were informed in writing prior to starting the questionnaire that survey completion implied consent for use of the data for research purposes. Participation was voluntary and uncompensated; there was no penalty for non-participation. In addition to residents’ baseline demographic data, survey data included type of training curriculum (CRP versus LRP) and parameters relevant to medical training, such as institutional type (university- versus community-based program). First implemented at the behest of the Health Ministry of Japan in 2008, the survey methods have been reported previously [10-12]. Briefly, prior to 2004, the Ministry of Health and Wealfare MHLW decided to establish a new mandatory 2-year rotation program for new graduates. In order to truly ensure that junior trainees would not be shuttled into specialty curricula, a Resident Education Committee was formed, which designated 98 competency items and 85 diseases or symptoms as mandatory for all young residents to experience.To assess whether these goals of generalism were being achieved, we used the same set of competency and case criteria for our study.

Main outcomes were self-reported confidence for 98 clinical competency items and number of cases experienced for 85 medical conditions. Confidence was recorded on a 4-point Likert scale as follows: 1 = very confident to perform independently; 2 = fairly confident to perform independently; 3 = no confidence to perform independently; and 4 = cannot perform. Number of cases experienced for 85 diseases commonly encountered during 2 years in residency training were recorded as follows: 1 = no cases, 2 = 1-5 cases, 3 = 6-10 cases, or 4 = ≥11 cases. Clinical confidence was dichotomized into ≤2 points versus ≥3 points for analysis, while case experience scores were dichotomized by ≥1 case versus no case experience. Case experience was further categorized into ≤2 points versus ≥3 points for multivariate logistic regression analysis, anticipating that many residents experienced at least 1 case for most diseases. All clinical competency items and diseases are detailed in the appendix (Appendix 1 and 2). Surveys were distributed via the administrative offices of individual residency programs. Participation in the survey was voluntary and residents received no compensation for participation.

Anonymized data were collected and descriptive statistics were used to explore the data. Chi-squared tests were used to compare high confidence in clinical ability by comparing the percent of residents reporting a confidence score of 1 or 2 in CRP versus LRP programs. Similarly, Chi-squared tests were used to compare the percent of residents reporting at least one case experienced of each clinical survey item. Considering that age and gender have previously been reported to influence work and learning experience of Japanese residents [13], and that it is reasonable to assume that opportunity for patient care experience will be predicated on institution size and type, multivariate logistic regression was subsequently performed on discrepant items from the univariate analysis, using gender, age, hospital size, and institutional affiliation as covariates. A level of p <0.05 was used to indicate significance; all statistical tests were two-tailed. Data were analyzed using IBM SPSS statistics software version 20.0 J (IBM, Tokyo, Japan). Ethical approval was obtained from the Research Ethics Committee of St. Luke’s International Hospital, Tokyo, Japan (approval code: 11-R211).

## Results

Table 1 summarizes demographic data of participating residents. From a total of the 7506 PGY2 residents practicing in Japan at the time of the study, 5052 residents replied to the survey (response rate, 67.3%). 3265 respondents (64.6%) were male; mean age of respondents was 28 (SD, 3) years. 3846 residents (76.1%) reported training in an LRP curriculum. Among LRP residents, 2078 residents (55.2%) were enrolled in a university-based program. Significantly more LRP residents reported training in a university hospital than a community hospital (p <0.001).

**Table 1:** Baseline demographic data of surveyed residents

	**LRP**	**CRP**	**p-value**
N	3846 (76.1%)	1206 (23.9%)	
Gender, male	2166 (64.9%)	1099 (64.0%)	0.554
Age	27.84 (64)	27.94 (64)	0.135
University hospital	1840 (55.2%)	584 (34.0%)	p <0.0001
Hospital beds	870 pita	529 pita	p <0.0001
Urban practice location*	2496 (74.8%)	1157 (67.4%)	p <0.0001

*Urban practice location defined as areas with an average number of physicians greater than the national average of 230.4 per 100,000 persons.

For clinical competency items, LRP residents reported lower confidence scores in 12 items (Table 2). In contrast, CRP residents reported lower scores in none of the items surveyed. Particularly discrepant items included “diagnosing pregnancy” and “IV placement and phlebotomy for pediatric patients”, for which LRP residents reported confidence scores of 46.7% and 70.2% versus 55.5% and 77.9% among CRP residents, respectively. After adjustment for gender, age, and institutional affiliation, lower scores remained significant for 10 of 12 items (Table 3).

**Table 2:** Confidence or experience items demonstrating statistically significant discrepancy (p < 0.05 on Chi square testing) between CRP and LRP residents

	**CRP**	**LRP**
Clinical competency items*		
Diagnosis of middle ear abnormality by otoscopy	60.7%	52.4%
Diagnosis of pregnancy	55.5%	46.7%
Pediatric IV placement and phlebotomy	77.9%	70.2%
Assessment of severity and acuity in ER setting	88.7%	83.1%
Diagnosis of prostate abnormality by rectal examination	60.6%	55.1%
Diagnosis of depression	59.9%	54.6%
Explanation to pediatric patients	85.5%	80.4%
Lumbar puncture	89.3%	84.4%
Appropriate pharmacotherapy for psychiatry patients	60.3%	55.8%
Diagnosis and treatment of shock	84.4%	80.5%
Spinal fluid analysis	82.3%	78.6%
Appropriate handwashing	100.0%	98.5%
Cases experienced**		
Pregnancy and delivery	99.8%	86.6%
Pediatric seizures	98.3%	89.0%
Pediatric asthma	97.6%	90.2%
Pediatric viral infection	99.2%	93.2%
Urologic disease		96.0%	90.8%
Keratoconjunctivitis	92.6%	89.6%	
Dislocation, subluxation, lower extremity sprain	97.6%	95.2%	
Somatoform disorder	99.3%	96.9%	
Allergic rhinitis	100.0%	98.2%	
Cellulitis	100.0%	98.6%	
Integration disorder syndrome	100.0%	98.6%	
Rheumatologic disorder	94.6%	97.1%	

*Reported as % number of responders rating confidence as 1 or 2 on a 4-point Likert scale.

**Reported as % number of responders reporting experience of ≥1 case.

**Table 3:** Adjusted odds ratios for items receiving higher scores among CRP residents

	**OR**		**OR 95% CI**			**p-value**
Clinical confidence items*					
Diagnosis of pregnancy	1.3	1.2	~	1.5	.000
Lumbar puncture	1.3	1.1	~	1.6	.011
Pediatric IV placement and phlebotomy	1.4	1.0	~	1.9	.029
Diagnosis of depression	1.4	1.1	~	1.6	.001
Explanation to pediatric patients	1.2	1.1	~	1.4	.002
Appropriate pharmacotherapy for psychiatry patients	1.2	1.1	~	1.4	.001
Spinal fluid examination	1.1	0.9	~	1.3	.403
Diagnosis of middle ear abnormality by otoscopy	1.3	1.1	~	1.5	.000
Diagnosis of prostate abnormality by rectal examination	1.1	1.0	~	1.3	.160
Assessment of severity and acuity in ER setting	1.1	0.9	~	1.4	.200
Diagnosis and treatment of shock	1.0	0.9	~	1.2	.640
Cases experienced**					
Pregnancy and delivery	3.4	2.9	~	3.9	.000
Pediatric seizures	1.2	0.9	~	1.6	.204
Pediatric asthma	1.2	0.9	~	1.6	.176
Pediatric viral infection	1.5	1.3	~	1.8	.000
Urologic disease	1.3	1.1	~	1.5	.000
Keratoconjunctivitis	1.2	1.1	~	1.4	.003
Dislocation, subluxation, lower extremity sprain	1.2	1.0	~	1.4	.010
Somatoform disorder	1.3	1.0	~	1.7	.039
Allergic rhinitis	1.2	0.9	~	1.5	.491
Cellulitis	1.1	0.8	~	1.6	.242
Integration disorder syndrome	1.4	1.1	~	1.8	.017
Rheumatologic disorder	1.1	0.8	~	1.4	.513

*Indicates adjustment for gender, age, size of hospital, and institutional type.

**Indicates case experience dichotomized into ≤5 cases vs. ≥6 cases experienced during training.

Regarding experience with diseases commonly encountered in primary care, LRP residents reported less experience with 11 diseases; OBGYN- and pediatrics-related items and cases were found to be especially low (Table 2). After adjustment for covariates (gender, age, and institutional affiliation), lower case experience remained significant for 8 of 11 diseases such as “Pediatric IV placement and phlebotomy (OR, 95%CI: 1.4,1.0 ~ 1.9), “Diagnosis of pregnancy (OR, 95%CI: 1.3,1.2 ~ 1.5) and so on. In contrast, CRP residents reported less case experience with only 1 disease, “rheumatologic disorder”. This did not retain significance after adjustment for clinically relevant covariates (OR, 95%CI: 1.1 ,0.8 ~ 1.4) (Table 3).

Despite conducting the survey at the end of the academic year (month 24 of a required 2-year curriculum), there were 775 residents (20.2%) in LRPs and 95 (7.9%) in CRPs who, for unclear reasons, reported ≤20 months of service (“non-compliers”). To address this, we conducted a sensitivity analysis in which non-compliers were excluded; we then conducted the same analysis for both competency and case experience. The results remained unchanged, suggesting that the impact of non-compliers is likely small.

## Discussion

This nationwide study evaluated self-reported clinical competency and number of cases experienced between residents in two different curricular programs completing their 2-year junior training requirements. This survey is the first to compare two different post-graduate training systems on a national level, yielding several important results.

First, proportionally more residents in university-based residency programs reported being trained in an LRP curriculum compared to residents at community hospitals. Community hospitals, typically offering a wider spectrum of primary care health services, have historically tended to offer broader rotation programs in order to meet unique community needs, such as those of rural and underserved areas. In contrast, university hospitals have historically focused on advanced, specialty-oriented care, as well as fostering the growth of basic science and translational research. Nonetheless, a large proportion of mid-career physicians from both university and community programs, regardless of specialty or geographic location, will leave their respective organizations in order to establish community-based primary care practices. This highlights the need for a modicum of robust generalist training for all physicians, regardless of training institution or practice location.

While the dichotomy between general and specialty medicine exists in health systems outside of Japan, as evidenced by increasing resident shifts from generalist to specialist post-graduate training programs in the United States, the benefits of specialists receiving initial training in an accredited general curriculum has been a well-established educational paradigm in both the US and UK, as well as numerous other countries [14-16]. In addition, health systems built around the central pillar of primary care gatekeeping may provide a buffer which allows both specialists and generalists to practice safely within their field of training. In contrast, the training environment in Japan has not typically offered or required standardized, longitudinal training in fields related to general practice (general internal medicine and family practice, specifically) prior to embarking on specialty training, especially among those training in university-based programs. The resulting ambiguity of the generalist-specialist dichotomy may obligate physicians to practice outside their scope of training, while furthering the divisions in perceived political and economic priorities between university- and community-based healthcare organizations.

Second, LRP residents were found to be both less confident and less experienced in a number of clinical competency items and diseases compared to residents training in the broader CRP curriculum, even after adjusting for institutional parameters, gender, and age. Though the majority of items were comparable, that CRP trainees were never associated with less confidence or case experience is a compelling finding. One reason for this may be that significantly more community hospitals offer CRP curricula; because community hospitals focus more heavily on primary-oriented care, residents in CRP likely have more opportunity to experience care of undiagnosed patients, as well as patient care in a wide variety of medical departments. However, the association between CRP training and both higher confidence and case experience continued to be significant, even after adjustment for institutional type. It is reasonable to assume that, apart from the site of training, broader curricula that allow for more access to multiple departments do indeed lead to more effective post-graduate medical training.

LRP residents’ reporting of areas of lower case experience and less clinical confidence appeared to be non-random and weighted towards basic care of relatively underserved populations in Japan. Suboptimal services included those for psychiatric patients (4 items) including diagnosis of depression, a fundamental skill in primary care. LRP residents also demonstrated lower confidence and less case experience in pediatrics (3 items) including viral infections and otoscopy, fundamental skills in general practice. Similarly, in OBGYN-related items (2 items), LRP residents reported less confidence with recognition and diagnosis of pregnancy. To put these findings in context, the large majority of items involving adult internal medicine were similar between CRP and LRP trainees. Albeit worrisome, these findings are unsurprising, as mandatory rotations through psychiatry, pediatrics, and obstetrics-gynecology were removed from LRP training requirements, thus limiting residents’ exposure to these items during emergency and community medicine rotations. While the argument may be made that pediatric and OB-GYN training is better restricted to those trainees specifically choosing careers in these fields, Japanese law and society place little restriction on subspecialists later returning to independent community medicine (“solo”) practices, of which women and children may comprise a substantial portion of the patient population. CRP training curricula may offer an additional safeguard to ensure that providers who do provide care to children and pregnant women have at least a modicum of training in these fields. This may be especially critical for Japan, which has experienced substantial shortages of pediatric and OB-GYN physicians over the last two decades [17,18]. This dearth has disproportionately affected rural and underserved areas, with international and domestic media reporting on several high-profile cases of catastrophic OB-GYN-related outcomes due to provider shortages [19,20].

Though the strength of this study primarily lies in its large numbers and impressive response rate, there are several issues that warrant discussion. First, these competency data are based on self-reported confidence assessment, and self-reported number of cases experienced. Though objectively registered outcomes would be ideal, given the national-level scope of this study, it was not feasible to implement a third-party observer-driven study design. It should be noted that, while there is much interest in objective and standardized assessments of cases experienced and competency in post-graduate medical training, these are currently not routinely assessed in the majority of Asian, North American, or European programs. While an element of bias may be introduced in our study, we expect this to be largely non-differential in nature between CRP and LRP participants. Second, we categorized training curricula as either CRP or LRP based on residents’ self-reported time spent in departmental rotations. However, as noted, a sizeable number of residents, especially those in LRP programs, reported training for less than the required 24 months, suggesting that some programs may be non-complaint with either curricular strategy. This may indicate a policy-level need for ensuring that standardization in training requirements is followed uniformly. Currently, there is no universally-accepted accrediting organization in Japan to ensure compliance with residency training standards; our data suggests a need for further investigation regarding compliance with national training guidelines. Should such an organization be established, the monitoring of objectively registered outcome data may be more feasible and would warrant evaluation to corroborate our results.

In addition, while many medical education systems around the world grapple with finding the optimal balance between specialty and general training, our results are most valid for the Japanese training context in which the survey was designed and implemented; country- and organization-specific needs and solutions addressing organizational structure, labor resources, work culture, as well as the economic and political dynamics of healthcare warrant careful consideration and thorough evaluation.

Nonetheless, our data strongly suggests that broader training curricula may effectively enhance confidence in general care, and raises interesting avenues for future research. Specifically, longitudinal research to evaluate whether higher confidence translates into measurable improvements in skill and outcomes, and whether these improvements last into mid-career, are needed to fully clarify the impact of training models on large-scale health quality improvement.

## Conclusions

Residents in limited rotation programs reported both less confidence and less case experience on clinical competency items and diseases compared to their peers in broader comprehensive rotation programs. To acquire and maintain crucial generalists skills needed later in physicians’ careers, especially those involving pediatrics and gynecology, residency training programs should consider ensuring that all residents receive standardized training in a wide variety of core departments, with particular attention to the care of women and children.

## Appendix 1: Self-reported confidence for 98 clinical competency items

1. Elicit patients' interpretative model of health/care

2. Systematic approach to patient medical history

3. Non-verbal patient communication

4. Gathering vital sign data

5. Describing skin findings

6. Diagnosis of arteriosclerosis by ophthalmoscopy

7. Diagnosis of otitis by otoscopy

8. Palpation of thyroid

9. Palpation of apex beat

10. Examination and assesment of heart sounds

11. Examination and assesment of wheeze

12. Examination and assesment of abdominal guarding

13. Diagnosis of prostate abnormality by digital rectal examination

14. Examination and assesment of early signs of pregnancy

15. Diagnosis of genital abnormality on bimanual examination

16. Examination and assessment of joint range of motion

17. Examination and assesment of meningeal signs

18. Examination and assesment of pediatric psychomotor developmental abnormalities

19. Evaluation and assesment of diagnostic criteria for depression

20. Diagnosis of bone fracture/dislocation/sprain

21. Diagnosis of RBC and WBC casts on urinalysis

22. Fecal occult blood testing and intepretation

23. Blood gas analysis and interepretation

24. Application and interepretation of leukocyte differential

25. Application and interepretation of blood chemistry

26. Application and interepretation of coagulation studies

27. Application and interepretation of other routine blood exams(glucose,electrolytes,BUN)

28. Application and interepretation of immunologic blood testing.

29. Analysis and intepretation of endocrinological examinations.

30. Gram stain examination and intepretation.

31. Cerebrospinal fluid examination and intepretation.

32. ECG examination and intepretation of arrhythmia.

33. Pulmonary function testing and diagnosis of pulmonary disease, including COPD.

34. Biliary ultrasound testing and assessmnent of bile duct.

35. Chest X-ray intepretation and dagnosis of silhouette sign.

36. Abdominal X-ray intepretation and diagnosis of ileus.

37. Chest CT intepretation and diagnosis of lung lesions.

38. Brain MRI intepretation and diagnosis of cerebral infarction.

39. Appropriate pre-operative handwashing.

40. Appropriate venous blood sampling.

41. Appropriate arterial blood sampling.

42. Blood type cross-matching analysis and intepretation.

43. Appropriate type and amount of IV for volume resuscitation.

44. Lumbar puncture

45. Urinary catheterization

46. Appropriate understanding of use and side effects of antibiotics

47. Performing local anesthesia and management of complications

48. Incision and debridement

49. Wound suturing

50. Management of post-operative complications

51. Management of pre-operative patient anxiety

52. Cardiac massage

53. Endotracheal intubation

54. Initiation and maanagement of ventilator

55. Cardiopulmonary resuscitation and AED use

56. Assessment of severity of ER patients

57. Diagnosis and management of shock

58. Patient- and family-centered commnication of terminal cancer diagnoses

59. Guidance and resource couselling for patients choosing home care.

60. Palliative care team participation (including WHO cancer pain treatment).

61. Consideration of both physical and psychosocial components of care.

62. Provision of information regarding medical costs, social services, and family couselling resources.

63. Taking appropriate informed consent.

64. Appropriate consultation with supervising phyisicians and specialists.

65. Provision of diabetic education.

66. Knowledge of insurance points for routine exams.

67. Understanding of role of social work and appropriate utilization.

68. Smoking cessation

69. Provision of patient education appropriate to knowledge and interest level

70. Evaluation and assessment for home care.

71. Utilization of community service resources and appropriate adjustment of treatment plans at discharge.

72. Understanding of role of community services and social wealfare institutions and appropriate utilization.

73. Evidence-based practice improvement including online searches.

74. Patient presentation skills during hospital conferences.

75. Patient charting (including discharge summaries) using the POS (Problem Oriented System) structure.

76. Understanding of research design and appropriate use of scientific articles.

77. Presentation at academic conferences.

78. Understanding and use of appropriate statistical analyses.

79. Understanding and implementation of patient safety paradigm.

80. Adherance to patient safety protocols.

81. Basic understanding of infectious disease control paradigms and protocols.

82. Rapid assessement of geriatric audiovisual and cognitive function.

83. Condunting of physical exams appropriate to geriatric patients' abilities.

84. Geriatric management appropriate to physical, mental, and social functioning.

85. Pediatric IV placement and phlebotomy.

86. Management of physical and psychosocial issues among pediatric patients.

87. Provision of appropriate explanations to pediatric patients.

88. Pharmacologic management of common psychiatric diseases.

89. Management of psychiatric pharmacotherapeutic complications.

90. Understading of role of mental health comedical staff, including psychiatric social workers (PSW), and appropriate utilization.

91. Appropriate utilization of communitymental health services.

92. Maintainence of confidentiality of patients information

93. Explanation of basic patient rights

94. Assistance with decision-making in appropriate patients.

95. Fromaulation of treatment plans (diagnosis, treatment, family and patients accountability)

96. Appropriate utilization of diagnostic and management guidelines and algorithms.

97. Participation in the training of younger physicians regarding clinical knowledge and skills.

98. Appropriate role modelling for younger physicians.

Scores were recorded on a 4-point Likert scale as follows: 1 = very confident to perform independently; 2 = fairly confident to perform independently; 3 = no confidence to perform independently; and 4 = cannot perform.

## Appendix 2: Number of cases experienced for 85 diseases commonly encountered during 2 years in residency training

1. insomnia

2. edema

3. lymphadenopathy

4. rash

5. fever

6. headache

7. dizziness

8. vision disorder, restricted vision

9. redness of the conjunctiva

10. chest pain

11. palpitations

12. dyspnea

13. cough/productive cough

14. nausea/vomiting

15. abnominal pain

16. abnormality of bowels (diarrhea/constipation)

17. back pain

18. limb paralysis

19. hematuria

20. dysuria/incontinence

21. anxiety/dysphoria

22. cardiopulmonary arrest

23. shock

24. disturbance of consciousness

25. cerebrovascular disease

26. acute heart failure

27. acute coronary syndromes

28. acute abdomen

29. acute gastrointestinal bleeding

30. trauma

31. acute intoxication

32. aspiration

33. burn injury

34. suicide attempt

35. anemia (iron-definciency/secondary anemia)

36. cerebrovascular disease (stroke/intracerebral bleeding/SAH)

37. eczema, dermatitis (contact/atopic)

38. urticaria

39. cellulitis

40. bone fracture

41. dislocations/sprains/tendinopathy

42. osteoporosis

43. spinal column disorder (disc herniation)

44. cardiac arrest

45. angina/myocardial infarct

46. cardiac dysrhythmia (tachyarrhythmia/bradyarrhythmia)

47. arterial disease (arteriosclerosis/aortic aneurysm)

48. hypertension (essential/secondary)

49. respiratory failure

50. respiratory tract infection (acute URI､ bronchitis､ pneumonia)

51. obstructive and restrictive pulmonary disease(bronchial asthma/bronchiectasis)

52. diseases of upper GI tract (esophageal varices/gastric cancer/peptic ulcer/gastritis/duodenal ulcer)

53. diseases of lower GI tract (ileus/acute appendicitis/ hemorrhoids/anal fistula)

54. hepatic diseases (virus hepatitis/acute and chronic hepatitis/cirrhosis/hepatic cancer/alcoholic liver disease/medication-related liver diseaes)

55. disease of diaphragm, abdominal wall, and peritoneum (peritonitis/acute abdomen/hernia)

56. Renal diseases(AKI/CKD/ESRD)

57. disease of urological tract(urolithiasis/UTI)

58. obstetrics (vaginal delivery/miscarriage/pre-term delivery/mastitis/puerperium)

59. male urologic disease(prostate disease/erectile dysfunction/testicular tumor)

60. metabolic disorders (DM/DM complications/hypoglycemia)

61. hyperlipidemia

62. ametropia(near-sighted/far-sighted/astigmatism)

63. keratoconjunctivitis

64. cataract

65. glaucoma

66. tympanitis

67. allergic rhinitis

68. Dementia(including secondary demantia)

69. Depression

70. schizophreniform disease

71. somatoform disorder/stress

72. viral infections (influenza/measles/rubella/chickenpox/herpes/mumps)

73. bacterial infections (Staphylococcu/MRSA/group A streptococcus/chlamydia)

74. tuberculosis

75. rheumatic gout

76. allergic disease

77. pediatric seizure

78. pediatric viral infections (measles､mumps､chickenpox､roseola､influenza)

79. pediatric asthma

80. geriatric nutritional disorders

81. geriatric disease (aspiration､falling､incontinence､bedsores)

82. death pronouncement

83. post-mortem documentation

84. CPC report(autopsy report)

85. referral documentation

Scores were recorded on recorded on a 4-point Likert scale as follows: 1 = no cases, 2 = 1-5 cases, 3 = 6-10 cases, or 4 = ≥11 cases.

## Competing interests

The authors declare that they have no competing interests.

## Authors’ contributions

Study design: SO, OT, GD, TF. Data collection: SO, OT, GD, TF and all members of the Ministry of Health, Labour and Welfare of Japan “Residency Training System and Career Path.” Statistical analysis: SO, OT, GD. First draft: SO. Critical revision of the manuscript: OT, GD, TF. Supervision and project guidance: TF. All authors read and approved the final manuscript.

## Pre-publication history

The pre-publication history for this paper can be accessed here:

http://www.biomedcentral.com/1472-6920/14/141/prepub
